# PPARG Drives Molecular Networks as an Inhibitor for the Pathologic Development and Progression of Lung Adenocarcinoma

**DOI:** 10.1155/2020/6287468

**Published:** 2020-04-26

**Authors:** Min Zhao, Xiaoyang Li, Yunxiang Zhang, Hongming Zhu, Zhaoqing Han, Yan Kang

**Affiliations:** ^1^School of Life Sciences and Biotechnology, Shanghai Jiao Tong University, Shanghai, China; ^2^State Key Laboratory of Medical Genomics Ruijin Hospital, Shanghai Jiao Tong University School of Medicine, Shanghai, China; ^3^Shanghai Institute of Hematology, Ruijin Hospital Affiliated to Shanghai Jiao Tong University School of Medicine, Shanghai, China; ^4^Department of Hematology, Ruijin Hospital Affiliated to Shanghai Jiao Tong University School of Medicine, Shanghai, China; ^5^Department of Respiratory Medicine, Shanghai Ninth People's Hospital, Shanghai Jiao Tong University School of Medicine, Shanghai, China; ^6^Department of General Surgery, Children's National Medical Center, Washington, DC, USA

## Abstract

Previous studies showed that low PPARG expression was associated with poor prognosis of lung adenocarcinoma (LA) with limited mechanisms identified. We first conducted a large-scale literature-based data mining to identify potential molecular pathways where PPARG could exert influence on the pathological development of LA. Then a mega-analysis using 13 independent LA expression datasets and a Pathway Enrichment Analysis (PEA) was conducted to study the gene expression levels and the functionalities of PPARG and the PPARG-driven triggers within the molecular pathways. Finally, a protein-protein interaction (PPI) network was established to reveal the functional connection between PPARG and its driven molecules. We identified 25 PPARG-driven molecule triggers forming multiple LA-regulatory pathways. Mega-analysis using 13 LA datasets supported these pathways and confirmed the downregulation of PPARG in the case of LA (*p* = 1.07*e*^−05^). Results from the PEA and PPI analysis suggested that PPARG might inhibit the development of LA through the regulation of tumor cell proliferation and transmission-related molecules, including an LA tumor cell suppressor MIR145. Our results suggested that increased expression of PPARG could drive multiple molecular triggers against the pathologic development and prognosis of LA, indicating PPARG as a valuable therapeutic target for LA treatment.

## 1. Introduction

Lung adenocarcinoma is one of the most common histological subtypes of Nonsmall-cell lung carcinoma that accounts for about 85% of all cases of lung cancer worldwide [1]. The overall 5-year survival rate of lung cancer is low even after surgical treatment (about 69.6%) [2]. Therefore, more effective strategies of therapy are necessary.

Locating on chromosome 3 (base pairs 12,287,485 to 12,434,356), PPARG encodes a member of the peroxisome proliferator-activated receptor (PPAR) subfamily of nuclear receptors—Peroxisome proliferator-activated receptor *γ* (PPAR*γ*), which have been shown to possesses an antagonistic function against LA (PMID: 22843091). However, so far, limited knowledge of this PPARG-inhibiting-LA mechanism is known [3]. On the one hand, it has been shown that the expression of PPARG was reduced in LA progression cells [4], and the low PPARG expression was strictly correlated with poor prognosis of stage IA LA [3]. On the other hand, increased expression of PPARG has been positively associated with a better survival rate of LA patients [4]. Ni et al.'s work showed that overexpression of PPAR*γ* could inhibit the drug resistance effect of gefitinib in the treatment of LA by reducing the proliferation of gefitinib-resistant cells [4]. Susaki et al. showed that PPARG could inhibit the tumorigenic potential of NR0B1 in LA [3]. However, more studies are needed to identify the underlying mechanism of the role that PPARG plays in the pathological development of LA.

To dissect the role of PPARG in LA at the genetic level, we employed Pathway Studio (http://www.pathwaystudio.com) knowledge database to undertake large-scale literature mining effort and integrated its results with an analysis of multiple LA expression datasets. We identified a set of PPARG-driven molecular triggers, possibly contributing to inhibition of the development of LA through a quantitative regulation. Our results might add new insights into the understanding of the LA-inhibition role of PPARG.

## 2. Materials and Method

This study is organized as follows. First, a large-scale literature-based data mining was performed to identify genes as the disease markers and the regulators of LA. Subsequently, regulations of PPARG on these LA genes were identified under the Pathway Studio environment. After that, a mega-analysis was performed using 13 independent LA gene expression datasets to test the expression changes of PPARG and the LA genes that were regulated by PPARG. Finally, a Pathway Enrichment Analysis (PEA) has been conducted to explore the functionality of the PPARG-driven molecular triggers, with protein-protein interaction (PPI) network built. All data and analysis results were organized in an excel file named as PPARG_LA, which is downloadable at http://www.gousinfo.com/database/Data_Genetic/PPARG_LA.xlsx.

### 2.1. Literature-Based Pathway Analysis

Assisted by using Pathway Studio (http://www.pathwaystudio.com), we conducted a large-scale literature-based functional pathway analysis to investigate the potential biological associations between PPARG and LA. Specifically, we identified the genes influenced by PPARG and also regulating LA to build the connections between PPARG and LA. Only relationships with polarity were selected within the Pathway Studio database. Each and all of the relations identified were supported by one or more references (1255 references in total; please refer to the worksheet “PPARG-LA Regulation Pathway” within the file PPARG_LA). In the PPARG_LA→PPARG-LA Regulation Pathway, the reference information supporting the relations identified in the PPARG-LA regulatory pathways was provided, including the types of associations, the number of underlying supporting references, and the sentences where these associations had been identified and described. The expression changes of PPARG and its driven genes involved in the pathways were tested using a mega-analysis approach described as follows.

### 2.2. Gene Expression Data Selected for Mega-Analysis

Following the initial search with “Lung adenocarcinoma”, 634 microarray expression datasets were identified on gene expression omnibus (GEO; https://www.ncbi.nlm.nih.gov/geo/) [5]. Subsequently, the following criteria were applied: (1) the organism used in the study was *Homo sapiens*; (2) the data type was microarray expression profiling; (3) the studies were limited to a comparison between LA and healthy controls; and (4) the original data and the corresponding format file were downloadable. A total of 13 datasets satisfied the inclusion criteria for the mega-analysis, which are listed in Table 1.

### 2.3. Mega-Analysis Models

For PPARG and the 25 genes involved in the PPARG-LA regulatory pathway, the log2 fold-change (LFC) of the gene expression level was used to indicate the effect size. Both fixed-effects and random-effects models were employed to investigate and compare the effect size (doi:10.1002/jrsm.12). The heterogeneity of the mega-analysis was analyzed to study the variance within and between different studies. In the case that the total variance (Q) was equal to or smaller than the expected between-study variance (df), the within − study variance percentage (ISq) = 100% × (Q − df)/Q was set at 0, and a fixed-effects model was selected for the mega-analysis. Otherwise, a random-effects model was selected. Q-p represents the probability that the total variance was only due to within-study variance. The current study presented all the mega-analysis results in the worksheet “mega-analysis” of the excel file PPARG_LA (http://www.gousinfo.com/database/Data_Genetic/PPARG_LA). The full name and description of related stats were given within “mega-analysis.” All analyses were performed using Matlab (version R2017a; https://www.mathworks.com/products/matlab.html). Here, we used the term “mega-analysis” instead of “meta-analysis” to reflect that the LFC of each gene was calculated from the original data instead of results within publications.

### 2.4. Multiple Linear Regression Analysis

A multiple linear regression (MLR) model was employed to investigate the possible influence of sample size, country of origin, and study date on the gene expression in the case of LA. *p* values were reported for each of these factors.

### 2.5. Pathway Enrichment Analysis and Protein-Protein Interaction Analysis

To test the functional profile of the genes involved in the PPARG-LA regulation pathway, a Fisher's Exact Test based pathway enrichment analysis (PEA; Pathway Studio: Find Pathways/Groups Enriched with Selected Entities) was conducted using Pathway Studio (version 12.1.0.9; http://www.pathwaystudio.com) against Gene Ontology (GO; http://geneontology.org) and Pathway Studio pathways. Statistics for the enriched pathways were provided, including false discovery rate (FDR) corrected *p*-value and Jaccard similarity.

Moreover, based on the PEA results, a protein-protein interaction (PPI) network was constructed. Two genes were recognized as connected if they were identified to play roles within at least one common pathway (from Pathway Studio Pathway Collection) or functional group (from GO groups).

## 3. Results

### 3.1. PPARG-LA Contradirectional Common Targets

**Figure 1:**
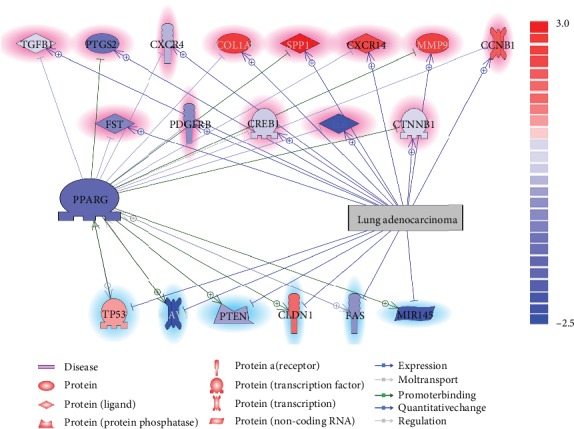
Contradirectional common targets of PPARG and lung adenocarcinoma according to literature that was also tested using expression data. Entities in blue represent a decreased expression level from the mega-analysis using 13 LA datasets; entities in red represent an increased expression level. Entities highlighted in blue (genes at the bottom of the figure) were literature implicated with a downregulation in the case of LA; highlighted in red (genes at the top of the figure) means they were upregulated. The polarity of the relationships was denoted as “-|” for negative effects and “-+>” for positive effects.

Pathway analysis has identified multiple molecules that were contradirectionally influenced by PPARG and LA, as shown in Figure 1. The expression levels of these genes, including PPARG, were tested in the mega-analysis and color-coded with the literature-based pathway (see the color bar in Figure 1).

According to previous literature reports, a total of 13 molecules upregulated in LA were negatively affected by PPARG (genes highlighted in red), and a total of six molecules suppressed in LA were stimulated by PPARG (genes highlighted in blue). The detailed information regarding the network presented in Figure 1 can be found in PPARG_LA⟶PPARG-LA Regulation Pathway with each network-related entry, including the type of the relationship, supporting references, and related sentences from the references where the relationship has been identified.

To note, five LA-unregulated genes presented increased expression levels in the mega-analysis using 13 LA datasets, including COL1A1, SPP1, CXCL14, MMP9, and CCNB1. The depression of these genes by PPARG could exert an anti-LA effect during its pathological development. On the other hand, four out of six LA-suppressed genes presented decreased expression levels in the mega-analysis, including three genes (CAV1, PTEN, and FAS) and one microRNA (MIR145). The activation of these molecules could be other pathways where PPARG inhibits the progress of LA.

Notably, PPARG presented a decreased expression level (*p* value = 1.07*e*-05, LFC = −0.48). This was consistent with previous studies [4]. Heterogeneity analysis showed that there was no significant between-study variance of PPARG expression levels (ISq = 0). Therefore, the fixed-effects model was used for the mega-analysis of PPARG. Moreover, MLR analysis suggested that none of the three parameters (sample size, country of origin, and study date) was a significant influence factor for the PPARG expression changes (*p* value > 0.070). For detailed info of the mega-analysis results of PPARG and other molecules, please refer to PPARG_LA⟶Mega-analysis.

### 3.2. PPARG-LA Regulation Pathway

**Figure 2:**
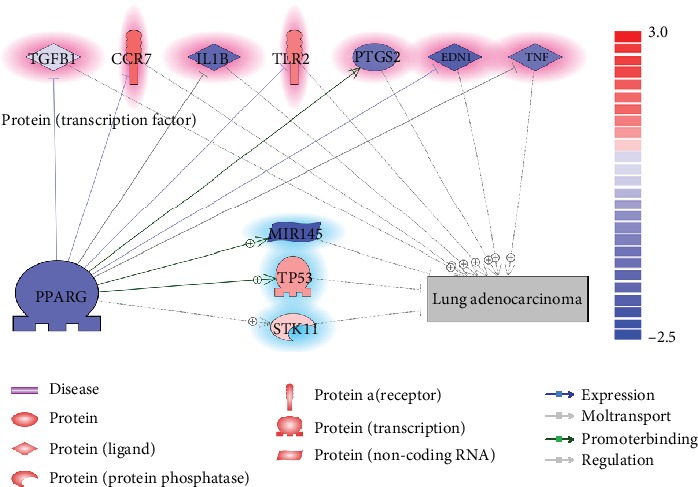
PPARG-LA regulatory pathways based on literature also tested by using expression data. Entities in blue represent a decreased expression level from the mega-analysis using 13 LA datasets; entities in red represent an increased expression level. Entities highlighted in blue (genes at the bottom of the figure) were literature implicated as an LA-inhibitor; highlighted in red (genes at the top of the figure) means they were LA promoter. The polarity of the relationships was denoted as “-|” for negative effects and “-+>” for positive effects.

We also identified a regulatory pathway through which PPARG may bar the pathological development of LA, as shown in Figure 2. According to literature reports, there were seven LA promoters (highlighted in red in Figure 2) deactivated by PPARG. Out of these molecular triggers, two genes presented increased expression levels in LA patients according to the mega-analysis results, including CCR7 and TLR2.

Literature data mining also revealed three LA inhibitors that could be activated by PPARG (see Figure 2; highlighted in blue). Only one of them, MIR145, presented a decreased expression level in the case of LA according to the results of mega-analysis, which was consistent with the negative-regulation relationship between LA and MIR145 that has been identified in literature data mining (see Figure 1). For more detailed information regarding the network presented in Figure 2, please refer to PPARG_LA⟶PPARG-LA Regulation Pathway.

### 3.3. PEA Results and PPI Network

To investigate the biological functions of the 26 genes within the PPARG-LA regulatory pathways (Figures 1 and 2), a pathway enrichment analysis was executed by using Pathway Studio. A total of 24 out of these 26 genes were shared among the top 10 most significantly enriched pathways (*p* value < 1.40*e*^−14^, *q* = 0.05 for FDR), which are presented in Table 2. The full 115 pathways and GO terms enriched with *p* value < 1.00*e*^−6^, which encompassed all 26 genes, were presented in PPARG_LA⟶PEA. Notably, a majority of the shared pathways highlighted by the PEA approach were related to cell proliferation and cell migration, which were implicated with LA.

**Figure 3:**
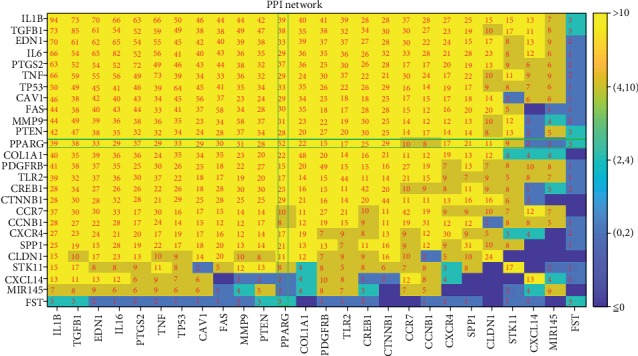
Protein-protein interaction networks among the 26 molecules involved in PPARG-LA regulatory pathways. The numbers between the two molecules are the number of pathways they shared out of the 115 pathways enriched by the 26 molecules.

Based on the significantly enriched pathways identified from PEA, a PPI network has been constructed, as shown in Figure 3 The number between two entities is the number of shared pathways out of the 115 pathways from PEA results. Please note that most of the molecules paly roles with all other molecules, suggesting the shared functionality of these molecules. Specifically, PPARG plays a role within 52 pathways and connecting with other molecules through 23.03 ± 12.90 pathways on average. This supports the literature-based relationship presented in the PPARG-LA regulatory pathways (Figures 1 and 2).

## 4. Discussion

In this study, we aimed at exploring the possible genetic mechanisms of the linkage between PPARG and better survival in LA. First, we conducted a large-scale literature-based data mining to construct functional PPARG-LA regulatory pathways (Figures 1 and 2). The 25 genes within these literature-based pathways were identified as connected to both PPARG and LA with polarity. Then, PEA was conducted to study the pathological functions of these 25 genes. After that, an LA expression data-based mega-analysis was performed to explore the expression levels of these 25 genes in the case of LA. Our results showed that PPARG could exert influence on both the development and progression of LA, which may add new insights into the understanding of the PPARG-LA association. We provided detailed descriptions of the PPARG-LA regulatory pathways as follows.

There were 19 molecules that have been identified as common targets but were contradirectionally regulated by PPARG and LA. These molecules have been reported to present altered expression levels in case of LA, and part of them (eight proteins and one microRNA) have been confirmed from the mega-analysis using 13 LA datasets employed in this study (please see Figure 1, the entities with body color matched with the highlighting color). With both literature and experimental data support, the pathways constructed with these molecules have high potential to be the PPARG**→**LA regulating channel. Specifically, the expression levels of five LA markers (i.e., COL1A1, SPP1, CXCL14, MMP9, and CCNB1) were significantly unregulated in LA patients [6–10], which were confirmed in the mega-analysis in this study. PPARG has been shown to inhibit hepatic stellate cell proliferation and COL1A1 expression in vitro and in vivo [11]. Similarly, the expressions of SPP1, CXCL14, MMP9, and CCNB1 have also been negatively regulated by PPARG [12–15]. On the other hand, PPARG could stimulate multiple molecules that inhibited by LA, including CAV1, PTEN, FAS, and MIR145. More descriptions of these PPARG regulations were provided in PPARG_LA**→**PPARG-LA Regulation Pathway. These pathways might partially explain the mechanisms of the development-blocking effect of PPARG on LA.

Moreover, PPARG has been shown to play roles in the prognostic pathway of LA (Figure 2). Both the literature data mining and the mega-analysis results support the suppression of four LA promoters (IL1B, PTGS2, END1, and TNF) and stimulation of one LA inhibitors, MIR145. As an LA tumor cell suppressor [16], MIR145 was found to be involved in multiple other types of cancers, including breast cancer [17], colon cancer [18], and acute myeloid leukemia [19]. However, as shown in Figure 1, in the case of LA, the expression levels of MIR145 will be downregulated, which also got confirmed from the 13-LA-datasets mega-analysis (see PPARG_LA**→**Mega-analysis). PPARG could activate the expression of MIR145 by directly binding to a PPAR response element in its promoter at 1207/-1194 bp from the transcription start site [20], which could be an important mechanism underlying the LA inhibition effect of PPARG.

PEA results indicated that each of the 25 PPARG-driven molecular triggers has at least two functional pathways shared with PPARG (see Figure 3). Most of these pathways were linked to cell proliferation, cell migration, and motility, indicating that PPARG may influence the LA development and progression through the cell metabolism and motivation pathways, which are important in the etiology of LA [21]. More interestingly, we see that PPARG was enriched in all top six pathways (see Table 2). These results support the functional association between PPARG and the 25 molecular triggers identified from the literature data mining (PPARG_LA→PPARG-LA Regulation Pathway).

To note, the expression of PPARG has been downregulated in LA, which is consistent with previous findings [4]. Therefore, our results suggest that the activation of PPARG could be a valid therapeutic strategy for the treatment of LA.

One of the limitations of this study was that the PPARG-driven LA pathways built only explored the genes connecting PPARG and LA. There could be more “bridge items” (e.g., functional class and compounds) to reveal more mechanisms underlying the PPARG_LA relation.

## 5. Conclusion

This study confirmed the downregulation of PPARG in the case of LA and revealed multiple pathways through which PPARG could play roles as a LA blocker. Our results shed light on the understanding of the PPARG-LA association, suggesting PPARG as a valuable therapeutic target for the treatment of LA.

## Figures and Tables

**Table 1:** The 13 LA expression datasets employed for mega-analysis.

Dataset GEO ID	Control (n)	Case (n)	Country	Study age	Sample Organism
GSE2088	30	9	Japan	11	Homo sapiens
GSE7670	28	27	Taiwan	13	Homo sapiens
GSE10072	49	58	USA	12	Homo sapiens
GSE31547	20	30	USA	9	Homo sapiens
GSE32863	58	58	USA	8	Homo sapiens
GSE32867	58	58	USA	8	Homo sapiens
GSE40791	90	94	USA	7	Homo sapiens
GSE43458	30	80	USA	7	Homo sapiens
GSE46539	92	92	Taiwan	4	Homo sapiens
GSE51852	4	49	Japan	6	Homo sapiens
GSE63459	32	33	USA	5	Homo sapiens
GSE68465	4	443	USA	5	Homo sapiens
GSE118370	6	6	China	1	Homo sapiens

**Table 2:** The top 10 genetic pathways enriched with 26 genes with the PPARG-LA regulatory pathways.

Name	GO ID	# of entities	Overlap	*p* value	Jaccard similarity
GO: response to oxygen levels	0070482	544	17	3.48*e*-18	0.031
GO: response to mechanical stimulus	0009612	334	14	2.46*e*-16	0.040
GO: response to inorganic substance	0010035	803	17	5.15*e*-16	0.021
GO: response to acid chemical	0001101	662	16	1.06*e*-15	0.024
GO: response to peptide	1901652	553	15	3.2*e*-15	0.027
GO: regulation of smooth muscle cell proliferation	0048660	215	12	3.62*e*-15	0.052
GO: response to hypoxia	0001666	424	14	3.85*e*-15	0.032
GO: positive regulation of cell migration	0030335	603	15	8.92*e*-15	0.024
GO: response to decreased oxygen levels	0036293	461	14	9.11*e*-15	0.030
GO: positive regulation of cell motility	2000147	630	15	1.4*e*-14	0.023

## Data Availability

The data in our study are available from the corresponding author upon reasonable request. The supplementary data and results were available at http://www.gousinfo.com/database/Data_Genetic/PPARG_LA.
